# Development of RT-RAA-CRISPR/Cas12a-Based Rapid Visual Detection Assay for Pigeon Rotavirus A

**DOI:** 10.3390/microorganisms14040732

**Published:** 2026-03-25

**Authors:** Cuiteng Chen, Yijing Hong, Zhongjun Tian, Mengyan Zhang, Zhen Chen, Chunhua Zhu, Lin Lin, Chunhe Wan, Yijian Wu

**Affiliations:** 1 College of Animal Science, Fujian Agriculture and Forestry University, Fuzhou 350002, China; chencuiteng@163.com (C.C.); 52506023045@fafu.edu.cn (Y.H.); 15121441882@163.com (Z.T.); zhangmengyan77@163.com (M.Z.); 2 Fujian Provincial Key Laboratory for Avian Diseases Control and Prevention, Institute of Animal Husbandry and Veterinary Medicine, Fujian Academy of Agricultural Sciences, Fuzhou 350013, China; zhenzhenssh@163.com (Z.C.); zchlxd80@163.com (C.Z.); linlin8220285@163.com (L.L.); 3 University Key Laboratory for Integrated Chinese Traditional and Western Veterinary Medicine and Animal Healthcare in Fujian Province, Fuzhou 350002, China; 4 Fujian Key Laboratory of Traditional Chinese Veterinary Medicine and Animal Health, Fujian Agriculture and Forestry University, Fuzhou 350002, China

**Keywords:** pigeon rotavirus A, CRISPR/Cas12a, reverse transcription recombinase-aided amplification, lateral flow strip

## Abstract

In recent years, pigeon rotavirus A (PiRVA) infection, an important emerging disease, has posed a major threat to the healthy development of the pigeon industry and public health. Therefore, developing an accurate, rapid and convenient detection method for this virus is vital for monitoring and early diagnosis of the disease. In this study, on the basis of the ORF sequence characteristics of the PiRVA VP6 gene, crRNA and reverse transcription recombinase-aided amplification (RT-RAA) primers were designed. On the basis of the CRISPR/Cas12a system, for the first time, the RT-RAA-CRISPR/Cas12a rapid detection method of PiRVA was established by combining RT-RAA and lateral flow strips. This method could specifically detect PiRVA, and there was no cross-reaction with other common viruses originating from pigeons. The minimum detection limit was 16.8 copies/μL, and the results of the intrabatch and interbatch repeated tests were consistent. Moreover, the method established in this study and the previously established common PCR method were used to analyse 56 clinical tissue samples from racing pigeons and domestic pigeons collected in 2025. The positive rates of racing pigeon and domestic pigeon samples detected by PCR were 17.6% and 12.8%, respectively, and the positive rates of racing pigeon and meat pigeon samples detected by the RT-RAA-CRISPR/Cas12a method were 23.5% and 17.9%, respectively, indicating that PiRVA infection occurs in both racing pigeon and domestic pigeon populations in China. In summary, the PiRVA RT-RAA-CRISPR/Cas12a detection method established in this study has good specificity, sensitivity, and reproducibility, and allows visualization of the results, which can be used for field applications. This study provides technical support for epidemiological surveillance and etiological research on PiRVA.

## 1. Introduction

Rotavirus (RV) is a double-stranded RNA (dsRNA) virus belonging to the order *Reovirales*, the family *Sedoreoviridae*, and the genus *Rotavirus* (https://ictv.global/taxonomy, accessed on 6 January 2026). The RV particles are icosahedral and consist of three layers, with a diameter of approximately 70–85 nm and no envelope. RV particles are wheel-shaped under an electron microscope [1]. The genome consists of 11 double-stranded RNA segments of different lengths, with fragment lengths ranging from 663 to 3302 bp. The genome encodes at least six structural proteins (VP1, VP2, VP3, VP4, VP6 and VP7) and five or six nonstructural proteins (NSP1, NSP2, NSP3, NSP4, and NSP5/NSP6) [2]. Two of the core proteins (VP1 and VP3) are directly associated with the genome, and VP2 makes up the core shell. The intermediate protein layer of the virion is made up of VP6. VP4 and VP7 make up the outermost protein layer [2]. In accordance with the antigenic properties of VP6 and the gel electrophoresis (PAGE) pattern of genomic RNA segments, RV can be divided into nine virus species or serogroups, A, B, C, D, F, G, H, I, and J [1]. According to current studies, rotavirus A (RVA) is the main epidemic and pathogenic strain of RV in humans and animals. This virus infects mainly young hosts, and the main clinical feature is diarrhea. RV was reported to have been isolated from pigeon droppings as early as the 1980s [3], but since then, relatively few studies have investigated RV in pigeons. It was not until 2016 that a G18P[17] genotype of pigeon rotavirus A (PiRVA) was discovered in pigeons with symptoms of vomiting, diarrhea, and liver necrosis in Australia [4]. In 2017, similar diseases were reported in domestic pigeons in Germany, Belgium, and Denmark. However, a retrospective analysis in 2018 indicated that this disease had actually existed in Europe for 30 years [5]. Rubbenstroth et al. subsequently reported that the PiRVA genotype G18P[17] was the main cause of young pigeon disease syndrome (YPDS) [6]. In addition, the USA [7], Poland [1,8] and Taiwan [9] reported cases of PiRVA infection in pigeons. As an important new disease that has emerged in recent years, PiRVA infection poses a major threat to the healthy development of the pigeon industry. Therefore, developing an accurate, rapid and convenient detection method for this virus is vital for monitoring and early diagnosis of the disease.

Clustered regularly interspaced short palindromic repeats (CRISPR) and CRISPR-related Cas proteins provide bacteria and archaea with adaptive immunity against viruses [10]. When bacteria are infected by a virus, they process the virus’s characteristic sequences and store them via CRISPR. When the same virus infects them again in the future, they can quickly recognize and resist it [11,12]. The CRISPR/Cas system is applicable not only to gene editing but also to the trans-acting cutting activity of Cas proteins, and it can also be used for the detection of pathogenic nucleic acids [13,14]. To detect the occurrence of trans-cleavage, one end of single-stranded DNA can be labelled with a fluorophore, such as FAM, and the other end can be labelled with a quenching group, such as BHQ1. If single-stranded DNA is cleaved, a fluorescent signal can be detected [15]. In addition, to facilitate onsite application and reduce reliance on instruments, researchers have used FAMs and biotin to label single-stranded DNA and developed a method to visually read the results via immunochromatographic strips [16,17]. The CRISPR/Cas system has low sensitivity for detecting nucleic acids and needs to be used in combination with nucleic acid amplification technology. Recombinase-aided amplification (RAA) is a method for rapid amplification of nucleic acids at constant temperature (37~42 °C). It mainly relies on three enzymes: recombinase, single-stranded DNA binding protein and strand-displacing DNA polymerase [18].

This study utilized the complementary advantages of the convenience of reverse transcription recombinase-aided amplification (RT-RAA) and the specificity of the CRISPR/Cas system in combination with lateral flow strips (LFSs). The RT-RAA-CRISPR/Cas12a visual rapid detection method for PiRVA was established for the first time. This method has the advantages of simple operation, low time consumption, good specificity, high sensitivity and no reliance on expensive instruments, providing technical support for the epidemiological monitoring and etiological research of PiRVA.

## 2. Materials and Methods

### 2.1. Virus and Samples

Viral strains and samples such as pigeon rotavirus A (PiRVA, FJ21109), pigeon paramyxovirus type 1 (PPMV-1, FJ2126), pigeon megrivirus (PiMeV, FJ2515), pigeon adenovirus 2 (PiAdV-2, FJ21125), pigeon parvovirus (PiPV, FJ2163), and pigeon circovirus (PiCV, FJ21152) were identified and preserved by the Institute of Animal Husbandry and Veterinary Medicine, Fujian Academy of Agricultural Sciences. The 56 samples (mainly from large-scale pigeon farms in Fujian Province) used for clinical detection were liver and intestinal tissues collected from dead pigeons sent for inspection in 2025. Viral nucleic acid was extracted via the EasyPure Viral DNA/RNA Kit (purchased from Beijing TransGen Biotech Co., Ltd., Beijing, China).

### 2.2. Design of RT-RAA Primers and crRNA

All the VP6 gene sequences with complete ORFs from the NCBI database were downloaded for analysis. Then, on the basis of the characteristics of the VP6 sequences and following the principle of constant-temperature amplification primer design, crRNAs and their corresponding 5 pairs of RT-RAA primers were designed. Among them, R1 and R2 were paired with the same upstream primer, F. The length of the designed RT-RAA primers was 31 bp, and the size of the amplification fragment was between 120 and 250 bp. The RT-RAA primers were synthesized by Sangon Biotech Co., Ltd, Shanghai, China. The PiRVA-specific crRNAs were synthesized by Shenzhen Yizhi Biotechnology Co., Ltd., Shenzhen, China. Specific information on the RT-RAA primers and crRNA is shown in Table 1.

### 2.3. Optimization of RT-RAA Reactions Optimization of the Reaction Conditions for RT-RAA

The reaction system (total volume: 20 μL) was prepared according to the instruction manual of the RT-RAA amplification reagent (purchased from Shenzhen Yizhi Biotechnology Co., Ltd., Shenzhen, China): 10 μL of rehydration buffer (2×), 0.5 μL of forward primer and reverse primer (20 μmol/L), 2 μL of template RNA, 5 μL of ddH_2_O, and 2 μL of starter (10×). The reaction system was placed in a constant-temperature water bath. First, the reaction primers were screened, and then the reaction temperature (35 °C, 36 °C, 37 °C, 38 °C, 39 °C, 40 °C) and reaction time (15 min, 20 min, 25 min, 30 min, 35 min, 40 min) were optimized. The reaction system with ddH_2_O added as the template was used as the negative control, and the reaction system with the positive template included in the kit was used as the positive control. The RT-RAA products were observed via 3% agarose gel electrophoresis and imaged via a gel imaging system. On the basis of the RT-RAA amplification results, the optimal primers and optimal reaction conditions were determined.

### 2.4. Optimization of CRISPR/Cas12a Reactions

In accordance with the instruction manual of the Cas12a reaction reagent (purchased from Shenzhen Yizhi Biotechnology Co., Ltd.), 20 μL of the following reaction mixture was prepared: 2 μL of Cas12a reaction buffer (10×), 1 μL of the Cas12a protein (1 μmol/L), 0.25 μL of the Cas12a reporter (4 μmol/L), 1 μL of crRNA (1 μmol/L), 1 μL of RT-RAA amplification product, and ddH_2_O were added to reach a total volume of 20 μL. Different combinations of final concentrations of Cas12a protein (5 nmol/L, 15 nmol/L, 25 nmol/L, 35 nmol/L, and 45 nmol/L) and final concentrations of crRNA (5 nmol/L, 15 nmol/L, 25 nmol/L, 35 nmol/L, and 45 nmol/L) were used, and reactions were carried out with the PiRVA-positive RT-RAA amplification products as templates. The reaction mixture was placed in a water bath and incubated at 37 °C for 30 min. After the reaction was completed, 2 μL of the product was removed, and 78 μL of diluent was added for dilution. The lateral flow test strips (purchased from Shenzhen Yizhi Biotechnology Co., Ltd.) were placed in the diluted reaction mixture, and the results of the strips were observed within 5 min. If one band appears on the strip, it is positive; if two bands appear, it is negative.

### 2.5. Specificity of RT-RAA-CRISPR/Cas12a Detection Reactions

Optimized RT-RAA amplification was performed on PiRVA, PPMV-1, PiMeV, PiAdV-2, PiPV, and PiCV, and then the RT-RAA amplification products were used as templates for detection under the optimized CRISPR/Cas12a reaction conditions. The reaction system with ddH_2_O added as the template was used as the negative control, and the reaction system with the positive template included in the kit was used as the positive control. The specificity of the method was examined by observing the results of the lateral flow test strip.

### 2.6. Sensitivity of RT-RAA-CRISPR/Cas12a Detection Reactions

The VP6 RNA of PiRVA (strain X-05, GenBank No. MN481635.1) was artificially synthesized by Sangon Biotech (Shanghai) Co., Ltd., Shanghai, China. The RNA was diluted 10-fold to a total of 9 dilutions ranging from 1.68 × 10^8^ to 1.68 × 10^0^ copies/μL. Using RNA diluted from 1.68 × 10^4^ to 1.68 × 10^0^ copies/μL as a template, RT-RAA-CRISPR/Cas12a detection was performed via the optimized detection method. The reaction system with ddH_2_O added as the template was used as the negative control, and the reaction system with the positive template included in the kit was used as the positive control. The sensitivity of the method was examined by observing the results of the lateral flow test strip.

### 2.7. Repeatability of RT-RAA-CRISPR/Cas12a Detection Reactions

Two concentrations of PiRVA RNA (1.68 × 10^6^ copies/μL and 1.68 × 10^3^ copies/μL) were used for the reproducibility test of RT-RAA-CRISPR/Cas12a. Each concentration was repeated 3 times. The reaction system with ddH_2_O added as the template was used as the negative control, and the reaction system with the positive template included in the kit was used as the positive control. Interbatch and intrabatch repeatability verification was conducted by observing the results of the lateral flow test strips.

### 2.8. Clinical Samples Detection

The established RT-RAA-CRISPR/Cas12a detection method was used to conduct PiRVA detection on 56 clinical tissue samples of racing pigeons and meat pigeons collected in 2025. Moreover, the common PCR method (upstream primer, PiRVAF: CAAACTGGAGGTATAGGCAACTTG; downstream primer, PiRVAR: TGCYACTCCAGGTGTCATATTTG; PCR product size, 777 bp) previously established in the laboratory and the qPCR method described in reference [5] were used for detection to evaluate the clinical application effect of this method. The PCR products with positive test results were sent to Sangon Biotech (Shanghai) Co., Ltd., for Sanger sequencing.

## 3. Results

### 3.1. Principle of the RT-RAA-CRISPR/Cas12a Visual Rapid Detection Method

**Figure 1:**
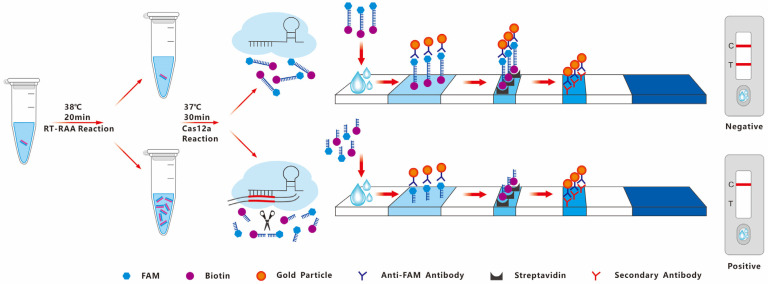
Schematic diagram of the RT-RAA-CRISPR/Cas12a visual rapid detection method for PiRVA.

A brief overview of the RT-RAA-CRISPR/Cas12a visual rapid detection method is shown in Figure 1. The RT-RAA reaction is carried out using the nucleic acid extracted from the sample to be tested as a template. If the sample to be tested contains the target gene, the target gene will be amplified in large quantities; otherwise, there will be no amplification. The Cas12a reaction system contains single-stranded DNA probes labelled with both fluorescein amide (FAM) and biotin. After the Cas12a reaction mixture was dropped onto the test strips, the FAM antibody linked to colloidal gold on the test strips bound to the single-stranded DNA probe, forming a detection probe of 5′-Gold-Ab-FAM-ssDNA-Biotin-3′. The streptavidin on the test (T) line captures the biotin-labelled test probe, forming a visible gold line. The secondary antibody on the control (C) line captures the FAM gold-labelled antibody, forming a visible gold line. If the test sample contains the target gene, Cas12a mediates crRNA to the target and cuts the target sequence. The transcutting activity of Cas12a is activated, the ssDNA probe is cut, and the FAM gold-labelled antibody cannot remain on the T line. There is no color band on the T line; only the C line has a color band, indicating a positive result. If the test sample does not contain the target gene, the transcutting activity of Cas12a cannot be activated, the single-stranded DNA probe remains intact, and the FAM gold-labelled antibody will remain on the T line by binding to the intact probe. The T line has a color band, and the C line also has a color band, indicating a negative result. If there is no band on the C Line or if neither the C Line nor the T Line has a band, the test strip is considered invalid.

### 3.2. Optimization of RT-RAA Reactions

**Figure 2:**
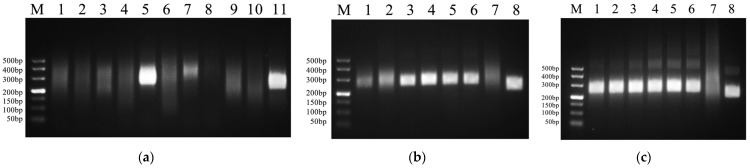
(**a**) RT-RAA primer screening results. M: DL 500 DNA Maker; 1: F/R1; 2: F/R1-H_2_O; 3: F/R2; 4: F/R2-H_2_O; 5: F3/R3; 6: F3/R3-H_2_O; 7: F4/R4; 8: F4/R4-H_2_O; 9: F5/R5; 10: F5/R5-H_2_O; 11: positive control of the kit. (**b**) Optimization results of RT-RAA reaction temperature. M: DL 500 DNA Maker; 1: 35 °C; 2: 36 °C; 3: 37 °C; 4: 38 °C; 5: 39 °C; 6: 40 °C; 7: ddH_2_O; 8: positive control of the kit. (**c**) Optimization results of RT-RAA reaction time. M: DL 500 DNA Maker; 1: 15 min; 2: 20 min; 3: 25 min; 4: 30 min; 5: 35 min; 6: 40 min; 7: ddH_2_O; 8: positive control of the kit.

In this study, five pairs of RT-RAA primers targeting PiRVA were designed and screened. The results of electrophoresis (Figure 2a) revealed obvious specific amplification bands for the primer pair F3/R3. The fragment size was 249 bp. Therefore, the primer pair F3/R3 was selected as the RT-RAA amplification primer for PiRVA.

When the reaction temperature was optimized, the temperatures were set to 35 °C, 36 °C, 37 °C, 38 °C, 39 °C and 40 °C, and the results are shown in Figure 2b. The target band can be amplified at 37 °C, 38 °C, 39 °C and 40 °C, but the amplification effect is greatest at 38 °C.

When the reaction time was optimized, the reaction times were set to 15 min, 20 min, 25 min, 30 min, 35 min, and 40 min, and the results are shown in Figure 2c. The target bands were amplified at 15 min, 20 min, 25 min, 30 min, 35 min and 40 min. After a reaction time of 20 min, the brightness of the bands changed little. To shorten the RT-RAA reaction time, the optimal reaction time was determined to be 20 min.

### 3.3. Optimization of Cas12a and crRNA Concentrations

**Figure 3:**
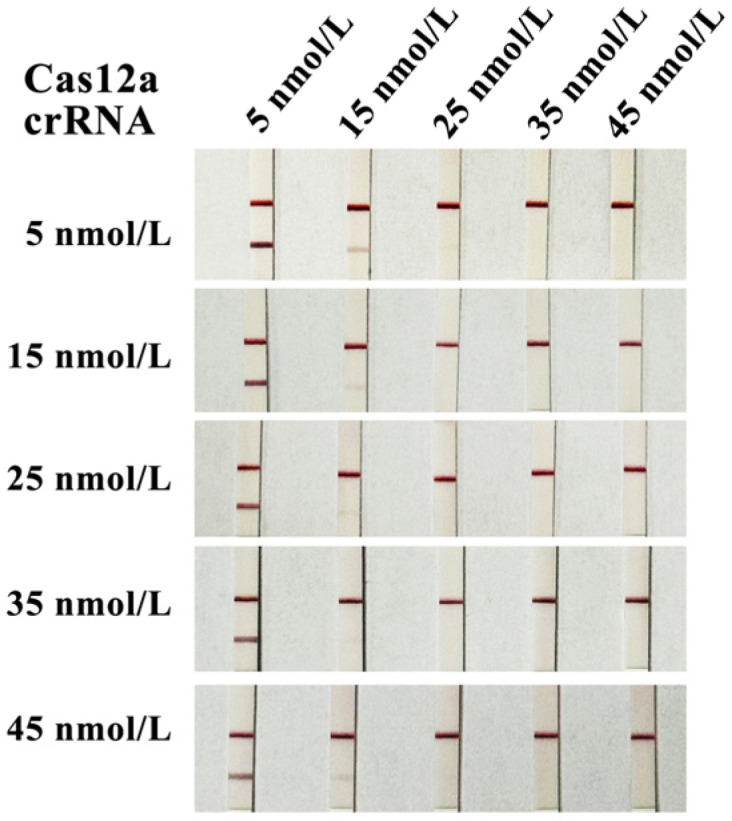
Optimization results of crRNA and Cas12a concentrations.

The test was conducted by setting different combinations of Cas12a protein and crRNA concentrations. One band on the test strip was considered positive, and two bands were considered negative. The results are shown in Figure 3. In the reaction system, when the final concentration of Cas12a protein was 25 nmol/L and the final concentration of crRNA was 15 nmol/L, the test strip results started to be positive. To save cost, the optimal final concentrations of the Cas12a protein and crRNA were determined to be 25 nmol/L and 15 nmol/L, respectively. Therefore, these concentrations of crRNA and Cas12a were used for detection in subsequent experiments. The optimized CRISPR/Cas12a reaction system (20 μL) consisted of 2 μL of Cas12a reaction buffer (10×), 0.5 μL of Cas12a protein (1 μmol/L), 0.25 μL of Cas12a reporter (4 μmol/L), 0.3 μL of CrRNA (1 μmol/L), 1 μL of RAA amplification product, and 15.95 μL of ddH_2_O.

### 3.4. Specificity, Sensitivity, and Repeatability

**Figure 4:**
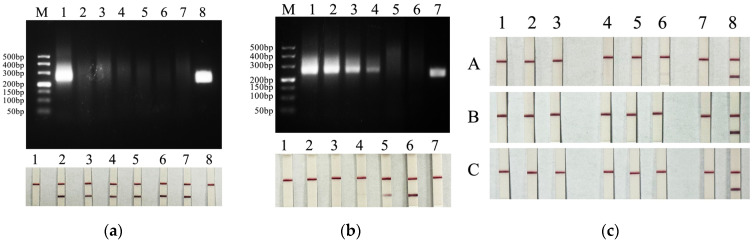
(**a**) Specificity test results of RT-RAA-CRISPR/Cas12a. M: DL 500 DNA Maker; 1: PiRVA; 2: PPMV-1; 3: PiMeV; 4: PiAdV-2; 5: PiPV; 6: PiCV; 7: ddH_2_O; 8: positive control of the kit. (**b**) Sensitivity test results of RT-RAA-CRISPR/Cas12a. M: DL 500 DNA Maker; 1–5: 1.68 × 10^4^–1.68 × 10^0^ copies/μL; 6: ddH_2_O; 7: positive control of the kit. (**c**) Repeatability test results of RT-RAA-CRISPR/Cas12a. 1, 2, 3: 1.68 × 10^6^ copies/μL; 4, 5, 6: 1.68 × 10^3^ copies/μL; 7: positive control of the kit; 8: ddH_2_O. A, B and C respectively represent three repeated experiments.

The results of the specificity test revealed (Figure 4a) that the established detection method could only specifically detect PiRVA and did not cross-react with other pigeon-derived viruses, such as PPMV-1, PiMeV, PiAdV-2, PiPV, and PiCV. The method has good specificity. As shown in Figure 4b, the minimum detection limit of the RT-RAA-CRISPR/Cas12a combined test strip detection method was 16.8 copies/μL. The results (Figure 4c) of the repeated tests of each concentration within the batch were consistent, and the results of the repeated tests of each concentration between batches were also consistent, indicating that the PiRVA detection method established in this study had good reproducibility.

### 3.5. Detection of Clinical Samples

**Figure 5:**
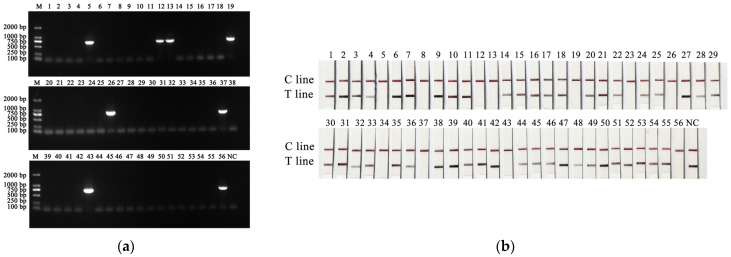
(**a**) The detection results of PCR for clinical samples. M: DL 2000 DNA Maker. (**b**) The detection results of RT-RAA-CRISPR/Cas12a for clinical samples. 1–56 clinical samples; NC: ddH_2_O.

The detection results of clinical samples are shown in Table 2. The results revealed that 8 samples were positive by the common PCR method (Figure 5a), the sequencing results of these 8 positive PCR products were also positive, and the percentage of PiRVA-positive samples was 14.3% (8/56). Eleven PiRVA-positive samples were detected via the RT-RAA-CRISPR/Cas12a method (Figure 5b), and the positive rate was 19.6% (11/56). Eleven PiRVA-positive samples were detected via the qPCR method (the Ct values of the positive samples were 21.09 ± 0.24, 33.12 ± 0.48, 25.21 ± 0.32, 24.40 ± 0.30, 23.06 ± 0.25, 32.24 ± 0.43, 21.18 ± 0.34, 32.06 ± 0.52, 23.39 ± 0.24, 19.00 ± 0.19, and 23.98 ± 0.33), with a positive rate of 19.6% (11/56). Moreover, the samples that were positive according to the common PCR method were also positive according to the RT-RAA-CRISPR/Cas12a method and qPCR method, and the positive samples detected by the RT-RAA-CRISPR/Cas12a method were the same as those detected by the qPCR method. Among the 8 samples that were positive in all three methods, 3 samples were from pigeons under 1 month of age, 2 samples were from pigeons under 2 months of age, and the other 3 samples were from pigeons under 3 months of age, 4 months of age and 6 months of age. Among the 8 samples that were positive in all three methods, 6 samples were from dead pigeons with clinical manifestations of enteritis. PiRVA positivity was detected in both racing pigeons and meat pigeons. The positive rates of racing pigeon and meat pigeon samples detected by the PCR method were 17.6% and 12.8%, respectively, and the positive rates of racing pigeon and meat pigeon samples detected by the RT-RAA-CRISPR/Cas12a method and the qPCR method were the same, at 23.5% and 17.9%, respectively. The detection results of the clinical samples revealed that the established RT-RAA-CRISPR/Cas12a method was reliable and that its sensitivity was greater than that of the conventional PCR method, with similar sensitivity to the qPCR method.

## 4. Discussion

RV is one of the main pathogens that causes severe acute gastroenteritis in young animals, infants and young children. The main clinical manifestations are vomiting, diarrhea, and dehydration. It is transmitted via the fecal–oral route. RV is a zoonotic pathogen that has a wide range of hosts and can be transmitted across species. It has been isolated from a variety of diseased animals. RV often becomes the cause of concurrent or secondary diarrhea diseases, posing a great threat to human health and the development of animal husbandry [19]. Early on, there were few reports of PiRVA infection. With the rapid development of the scale and intensification of the pigeon industry and the increase in the total amount and density of farming, an increasing number of reports of PiRVA infection have been published in recent years. Coupled with the frequent trade and circulation of pigeons, such as pigeon races and fancy pigeon exhibitions, these activities not only increase the risk of PiRVA transmission among pigeons but also provide a favourable environment for the recombination of the PiRVA virus, increasing the risk of loss of prevention and control of this disease. It has been reported that after exhibitions of fancy pigeons in Germany, the pigeons that participated in the exhibition broke out with a disease caused by PiRVA infection [20]. Other studies have shown that PiRVA has a relatively high prevalence among exhibited pigeons, indicating that exhibitions are a risk factor for the spread of this pathogen [21]. As an important emerging disease, PiRVA infection poses a major threat to the healthy development of the pigeon industry and public health. PiRVA infection, a newly emerging disease, has posed a considerable threat to the healthy development of the pigeon industry. Therefore, establishing a rapid detection method for this virus is vital for monitoring and early diagnosis of the disease.

Nucleic acid detection technology based on the CRISPR/Cas12a system, which has the advantage of high specificity, has been widely applied in pathogen detection in humans [22] and livestock and poultry, such as cattle [23], pigs [24], chickens [25], ducks [26], and geese [27], and is changing the traditional method of viral nucleic acid detection. It is a brand new viral nucleic acid detection technology. Combined with methods such as isothermal amplification, the CRISPR/Cas nucleic acid detection system can amplify signals through transcutting activity, reducing the requirements for instruments and making onsite specific and rapid detection a reality. Compared with conventional diagnostic methods, the CRISPR/Cas system is highly suitable for developing higher-performance, onsite rapid detection methods, which have greater advantages when resources are limited. This study established a rapid detection method for PiRVA based on the CRISPR/Cas12a system combined with RT-RAA and LFS. This method is simple to perform and takes a short time. The RAA reaction takes 20 min, the CRISPR/Cas12a reaction takes 30 min, and the results can be observed within 5 min with the test strip. No expensive instruments are needed. It has strong specificity and can detect only PiRVA without cross-reaction with other common pathogens in pigeons. It has high sensitivity, with a detection limit of 16.8 copies/μL, which is higher than that of the common PCR method. The results of three repeated experiments show that this method has good repeatability, and the results are visualized. It is very suitable for on-site detection and provides technical support for epidemiological surveillance and etiological research on PiRVA.

YPDS is a common seasonal acute intestinal disease in young pigeons. Its clinical manifestations include vomiting, diarrhea, lethargy, anorexia, and weight loss. This disease spreads rapidly, with an incidence rate as high as 100%, while the mortality rate ranges from zero to over 40% [28]. After pigeons suffer from diarrhea, their production performance declines, and they may even die. Diarrhea in racing pigeons often leads to a decline in their flying ability and loss of economic value, which is one of the major diseases that severely affects the pigeon industry. Previous studies have shown that the YPDS is caused by a variety of factors. Some pathogens, such as pigeon circovirus [29] and pigeon adenovirus [30], have been proposed, but none of them can replicate the disease experimentally. Rubbenstroth et al. discovered a novel variant of PiRVA in European domestic pigeons with clinical manifestations of diarrhea, vomiting, liver necrosis and sudden death. Through animal infection experiments, they demonstrated that PiRVA infection could cause diarrhea and death in pigeons. Therefore, they suggested that PiRVA was the main pathogen of disease syndrome in young pigeons [5,6]. The test results of the clinical samples in this study revealed that most of the PiRVA-positive samples were from dead pigeons with clinical manifestations of enteritis. Although the sources of clinical samples tested in this study were limited, the test results revealed that PiRVA infection occurs in both racing pigeon and meat pigeon populations in China and that pigeons under 6 months of age could all be infected with PiRVA. To determine whether there are differences in clinical symptoms among pigeons of different ages after being infected with PiRVA, further research is needed.

The meat pigeon farming industry has developed over several decades and has become the fourth largest poultry farming industry after chicken, duck and goose. Currently, China has become the world’s largest producer of meat pigeons, with an annual output of approximately 680 million pigeons, accounting for approximately 80% of the global output [31]. According to statistics from the Chinese Racing Pigeon Association, the total number of pigeon leg rings issued nationwide in China exceeded 30 million in 2025, and the pigeon racing industry is developing rapidly. Although the industry has broad prospects, the pigeon farming industry still faces multiple challenges, among which the pressure of disease prevention and control is one of the most important challenges. The timely monitoring and rapid diagnosis of diseases are greatly dependent on detection methods. The rapid detection method for pigeon group rotavirus RT-RAA-CRISPR/Cas12a established in this study only includes constant temperature amplification and CRISPR/Cas12a reaction processes. The process is simple, and only simple instruments such as pipettes and thermostat water baths are needed, without the need for expensive instruments such as PCR machines, gel imaging systems, and fluorescence quantitative PCR machines. It can be operated in most ordinary grassroots laboratories. Compared with traditional molecular biology methods, although this method currently does not have a significant advantage in terms of reagent costs, with the development of technology, the cost of reagent production will continue to decrease. It is believed that nucleic acid detection technology based on the CRISPR/Cas system will become an important development direction of the next generation of nucleic acid detection.

## 5. Conclusions

In conclusion, we first developed a rapid and accurate RT-RAA-CRISPR/Cas12a visual detection method for PiRVA. This method has excellent specificity, sensitivity and repeatability and can be used for rapid onsite detection, providing technical support for the diagnosis, epidemiological monitoring and etiological research of PiRVA infection. We also confirmed that PiRVA infection occurs in both racing pigeon and meat pigeon populations in China.

## Figures and Tables

**Table 1:** RT-RAA primers and crRNA sequence information.

Name	Sequence (5′-3′)	Position
PiRVA-RAA-F	CCTCAAGCGGCACCATTTCCAAATCACGCGA	925–955
PiRVA-RAA-R1	ATTAAGCAACTCAGTCCAATTCATACCTGCT	1086–1116
PiRVA-RAA-R2	TCAGTCCAATTCATACCTGCTGGAAACACTG	1076–1106
PiRVA-RAA-F3	CTAGATATGGCACGTTGGCCGCCCGTAATTT	824–854
PiRVA-RAA-R3	CTACTGGAATCGCGTATTCTTGTCTCAAACC	1042–1072
PiRVA-RAA-F4	CCAAATCACGCGACAGTTGGTTTGACACTAA	943–973
PiRVA-RAA-R4	CCAATTCATACCTGCTGGAAACACTGGTCCT	1071–1101
PiRVA-RAA-F5	CAATTGTAACAGGTTTGAGACAAGAATACGC	1031–1061
PiRVA-RAA-R5	TGTAAATATGCGTTGCAAGTTGTCTTCTCTA	1131–1161
PiRVA-crRNA	UAAUUUCUACUAAGUGUAGAU AGACAAGAAUACGCGAUUCCAGU	1048–1070

**Table 2:** Detection results of clinical samples.

Sample Source	Number	PCR	Positive Rate (%)	RT-RAA-CRISPR/Cas12a	Positive Rate (%)	qPCR	Positive Rate (%)
Racing Pigeons	17	3	17.6	4	23.5	4	23.5
Meat Pigeons	39	5	12.8	7	17.9	7	17.9
Total	56	8	14.3	11	19.6	11	19.6

## Data Availability

The original contributions presented in this study are included in the article. Further inquiries can be directed to the corresponding authors.

## Abbreviations

The following abbreviations are used in this manuscript:

PiRVA	Pigeon rotavirus A
RV	Rotavirus
RT-RAA	Reverse transcription recombinase-aided amplification
CRISPR	Clustered regularly interspaced short palindromic repeat
